# A unique spontaneously immortalised cell line from pig with enhanced adipogenic capacity

**DOI:** 10.1038/s41538-025-00413-y

**Published:** 2025-04-20

**Authors:** Thomas Thrower, Susanna E. Riley, Seungmee Lee, Cristina L. Esteves, F. Xavier Donadeu

**Affiliations:** Division of Translational Bioscience, The Roslin Institute and Royal (Dick) School of Veterinary Studies, University of Edinburgh, Edinburgh, UK

**Keywords:** Mesenchymal stem cells, Fats, Cell growth

## Abstract

Cultivated meat promises to address some of the pressing challenges associated with large-scale production of animals for food. An important limitation to realising such promise is the lack of readily available cell lines that can be expanded robustly for scale-up culture while maintaining the capacity to differentiate into tissues of interest, namely fat and muscle. Here, we report a porcine mesenchymal stem cell line (FaTTy) which, uniquely, upon spontaneously immortalisation acquired enhanced adipogenic efficiency, close to 100%, that has now been maintained for over 200 population doublings. FaTTy is able to differentiate with high efficiency in both 2D and 3D contexts and produces mature adipocytes upon prolonged differentiation. Moreover, FaTTy adipocytes display fatty acid profiles largely similar to native pig fat but with higher monounsaturated-to-saturated ratios. FaTTy displays minor aneuploidy, characterised by lack of Y chromosome, and lacks typical genetic or functional properties of tumorigenic cells. These highly distinctive characteristics, together with its non-genetically modified nature, make FaTTy a very attractive, potentially game-changing resource for food manufacturing, and particularly cultivated meat.

## Introduction

Pig farming provides the second-largest source of meat consumed worldwide. With global demand for meat set to grow during the next few decades, the sustainability of intensive pig production is under severe threat due to financial, environmental, and animal welfare concerns^1,2^. Cultivated meat promises to address some of the challenges associated with intensive animal farming by using cell culture approaches to reduce the need for animals for sourcing meat. A significant limitation to the realisation of such promise is the lack of suitable cell sources for large scale production of cultivated meat. In particular, progenitor cell lines are required from livestock species such as pig, that can be expanded and differentiated into meat-relevant tissue lineages with high efficiency under strictly defined conditions, including in the absence of animal-derived products^3,4^_._

Animal fats are key sensory components of food as they provide characteristic taste and texture as well as essential nutrients and energy. Therefore, reliably growing fat cells (adipocytes) at scale from livestock is a pre-requisite for industrial cultured meat manufacture. Natural progenitor cells derived from body tissues, also known as pre-adipocytes, adipose stem cells, or mesenchymal stem/stromal cells (MSCs), are the most common source of in vitro-grown adipocytes^4^. Other sources of in vitro fat such as pluripotent stem cells (PSCs) and de-differentiated fat cells (DFAT)^5^ are relatively amenable to scaling up applications but have important disadvantages in that they are less characterised and are technically more challenging to work with than natural progenitor cells (reviewed in Yuen et al.^3^). Yet, while easy to harvest, expand and differentiate in culture, primary fat progenitors have a limited replicative lifespan and exhibit variable adipogenic capacity. This variability is influenced by numerous factors including animal and tissue source. Additionally, their adipogenic potential diminishes with continuous passaging, collectively limiting their utility for industrial-scale in vitro meat manufacture^6–12^. Cell immortalisation has been used to address these limitations. MSC lines with extended proliferation capacity have been generated from pig and chicken through overexpression of TERT and/or specific cell cycle regulatory genes, however, these cells displayed limited adipogenesis, defeating the purpose of immortalisation^13,14^. Recently, an MSC line was reported in pig^15^ able to maintain its adipogenic capacity over time, however, the use of the oncogene SV40Tag for immortalisation makes this line far from ideal for cultivated meat purposes due to a lack of history of safe use in food.

Non-genetically engineered cell lines would be preferable from a regulatory perspective. To be useful for food manufacture, such cells would need to be amenable to scaling-up growth and differentiation, with sustained high adipogenic capacity, and genomically stable without transformation capacity. In that regard, progress has recently been reported in chicken with the generation of spontaneously immortalised fibroblast lines amenable to suspension, serum-free culture, and with the capacity to form adipocytes-like cells^16^. The adipocyte-like cells, however, showed limited fat production compared to fibroblasts following expansion in bioreactors.

Therefore, there remains a critical need for robust adipocyte cell lines for industrial cultivation of animal fat, which exhibit both sustained proliferation capacity and consistently high fat production. Here, we present a novel pre-adipocyte line (FaTTy) derived via serial passaging and spontaneous immortalisation of adipose-derived MSCs from pig which, highly uniquely, has been expanded to over 200 population doublings (PDs), with enhanced levels of adipogenesis, close to 100% efficiency, maintained throughout. Moreover, upon extended differentiation in vitro, FaTTy produces mature cells akin to white adipocytes, with lipid profiles comparable to native fat. Finally, FaTTy shows neither major chromosomal abnormalities nor transformation capacity in vitro, thus offering considerable high promise for use in cultivated meat manufacture.

## Results

### Derivation of a spontaneously immortalised MSC line with enhanced adipogenic capacity

**Fig. 1:**
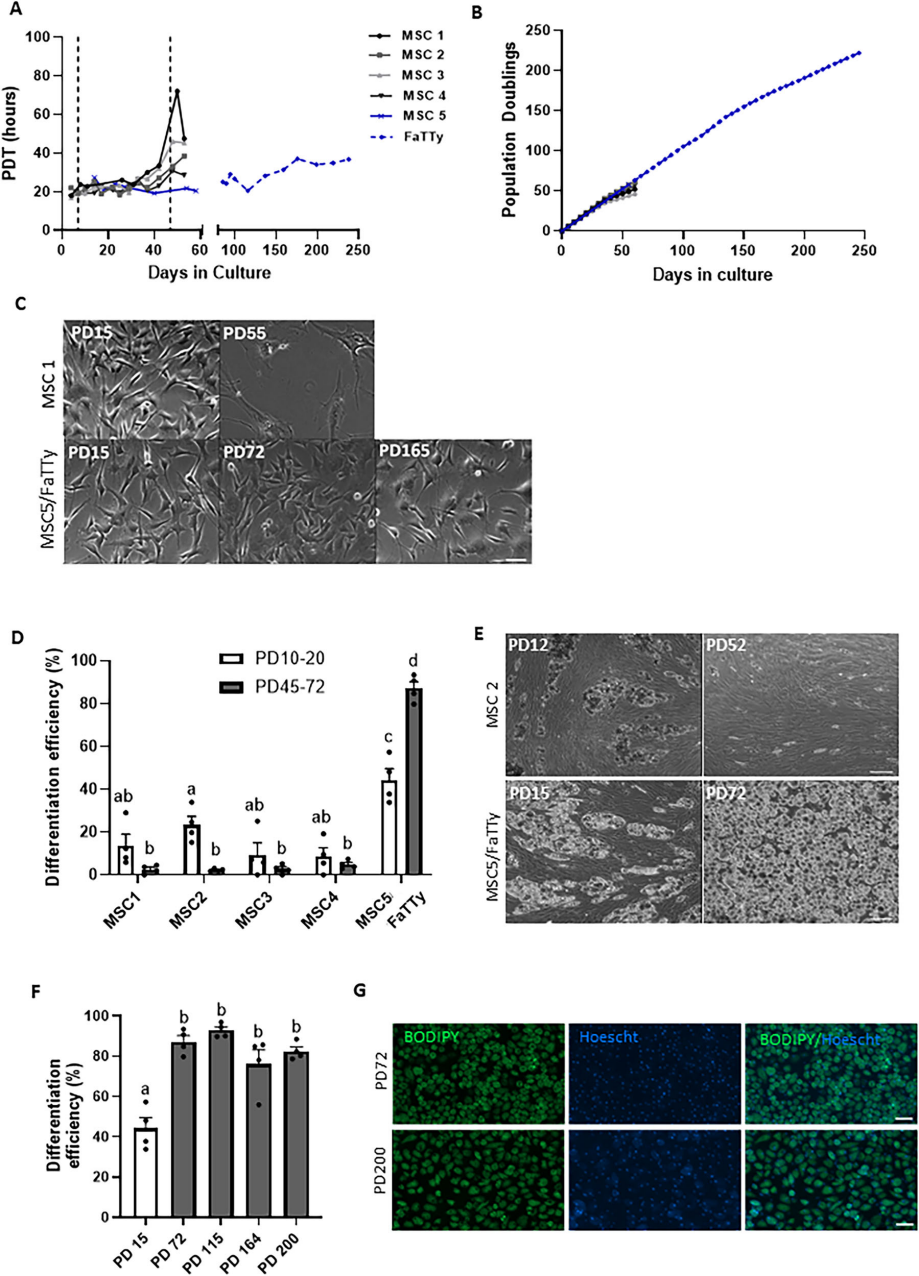
Derivation of the highly adipogenic cell line, FaTTy. **A**, **B** Growth profiles, represented by (**A**) population doubling times (PDTs) and (**B**) population doublings (PDs), of various MSC lines and FaTTy during extended culture. MSC5 corresponds to the FaTTy progenitor line. MSC1-4 were not cultured beyond 60 days as they had become senescent. Dashed lines in (**A**) indicate time points at which cells were assessed for adipogenic capacity (shown in **D**). **C** Representative brightfield images of MSC1 and MSC5/FaTTy lines (upper and lower rows, respectively) at different PDs, showing MSC1 cultures, but not FaTTy, becoming senescent with serial passaging, i.e. by PD55. Scale bar = 50 µm. **D** Adipogenic efficiency (defined by the proportion of cells staining positive for BODIPY after an 11-day differentiation) of various MSC lines and FaTTy at two different PDs, specifically PD9/53, PD9/58, PD6/46, PD6/52 and PD15/72 for each of MSC1,2,3,4 and 5/FaTTy, respectively (*n* = 4 images analysed per cell line and PD). **E** Representative brightfield images of MSC2 and FaTTy lines (upper and lower rows, respectively) at two different PDs after an 11-day differentiation, showing a distinct increase in differentiation capacity of FaTTy with time in culture. Scale bar = 100 µm. **F**, **G** Differentiation efficiency values (**F**) with representative fluorescent adipocyte images (**G**) obtained from FaTTy cells at different PDs following incubation with adipogenic media. Green = lipid (BODIPY); Blue = nucleus (Hoescht 33452). Where shown, bars represent mean + SEM. In (**D,F**) significant mean differences (*p* ≤ 0.05) are denoted by different letters (abcd).

While growing MSC lines harvested from five different piglets (MSC1-5, Fig. 1), we noticed that most lines (MSC1-4) maintained population doubling times (PDTs) of 20–24 h for about 30–40 days before gradually slowing down and showing signs of senescence, such as body cell enlargement and flattening. In contrast, cells from the MSC5 line (also known as FaTTy precursor) retained growth characteristics past the expected Hayflick limit of 40–60 PDs^17^, without apparent morphological signs of crisis, that is, without changes in their normal fibroblastic morphology (Fig. 1A-C). MSC5 was considered to be immortalised once it reached 60 PDs (equivalent to 57 days in culture) and was hereafter referred to as FaTTy. We found that adipogenic efficiency (defined by the proportion of cells staining positive for BODIPY after an 11-day differentiation) was variable initially among lines MSC1-4 (10 to 40%) and, in general, decreased markedly (to <5%) following serial passaging (Fig. 1D, E, and Supplementary Fig. 1), consistent with previous data^8,9,18,19^. Uniquely, in the case of MSC5/FaTTy, adipogenic efficiency actually increased during culture, with average efficiencies ( ± SEM) of 87 ± 5% at PD72 compared to 44 ± 3% in parental MSC5 cells at PD15.

Upon continued expansion, we observed a slight decrease in the growth rate of FaTTy from about 22 to 36 h between PD140 and PD190 (Days 140-200), followed by a period of growth stabilisation (Fig. 1A, B). This was associated with a slight enlargement of FaTTy cells which remained fibroblast-like nonetheless (Fig. 1C, lower panel). Most remarkably, these cells maintained their high adipogenic capacity throughout, as evidenced by differentiation efficiencies of 76–93% observed consistently across up to PD 200 (Fig. 1F, G). Moreover, under 2D, FaTTy was able to differentiate in a variety of serum-free conditions (albeit with slightly less efficiency than in the presence of serum), and could readily generate adipocytes in as little as four days (Supplementary Fig. 2A). FaTTy also differentiated in 3D using alginate hydrogels^9^ whereby ‘steaks’ of fat were produced (Supplementary Fig. 2B). Considered together, these properties confer FaTTy clearly distinct advantages for prospective cultivated meat applications. Subsequent attempts at re-deriving FaTTy-like cells from their original MSC progenitors yielded new lines showing increased differentiation efficiencies with time in culture, however this was not sustained past about 10 weeks, whereby cells displayed a significant reduction in differentiation potential (Supplementary Fig. 3), as well as slowing down in growth, both indicative of senescence.

From the above results, we concluded that the emergence of FaTTy could be attributed to a rare event, and that FaTTy was doubly unique in that it resulted from spontaneous immortalisation of a progenitor population and displayed increased adipogenic potential over time.

### FaTTy cells are karyotypically and transcriptionally distinct from parental MSCs while showing no transformation potential

**Fig. 2:**
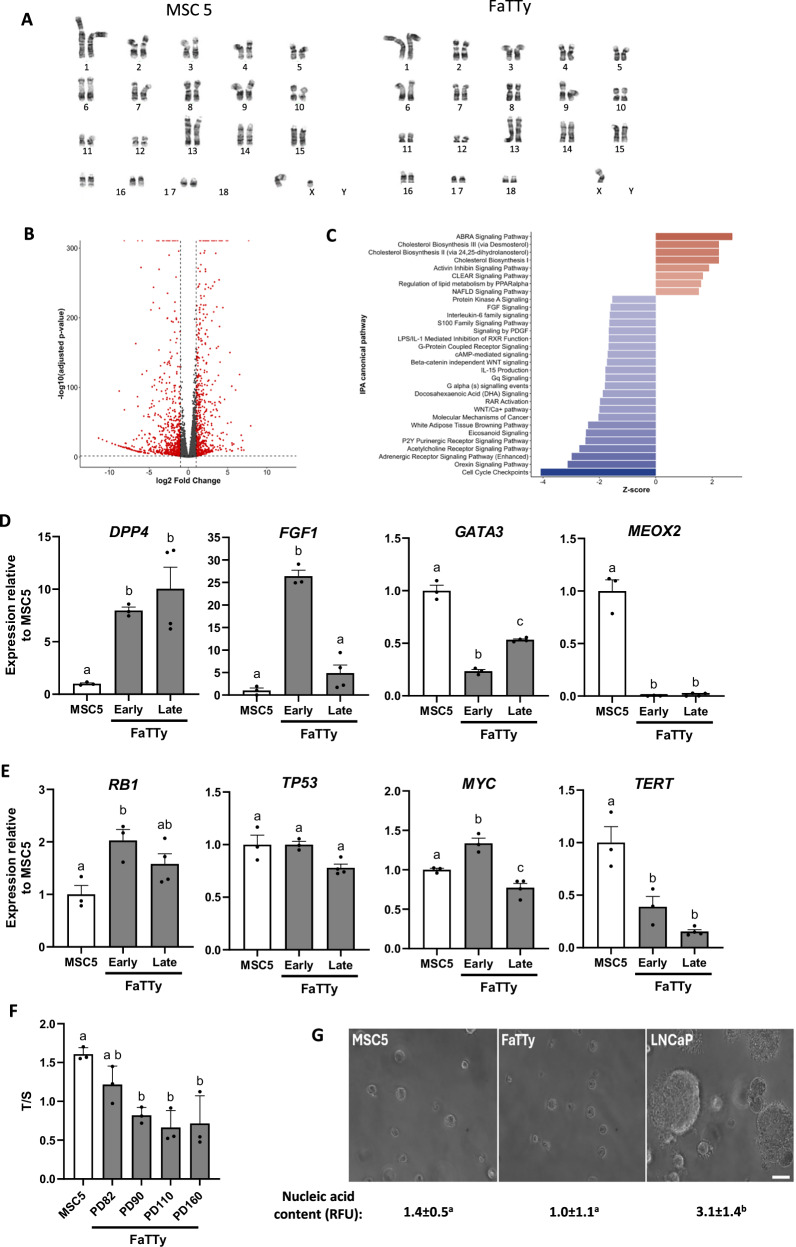
Karyotype, transcriptome and transformation potential of FaTTy cells. **A** Representative G-banded metaphase spreads of parental (PD15) and FaTTy (PD72) cells. Y chromosome was absent from 19 out of 20 FaTTy spreads analysed. **B** Volcano plot showing all unique transcripts (each represented by a dot) obtained by RNA sequencing. Genes differentially expressed in FaTTy (PD72) relative to parental (PD15) cells ( ≥ 2-fold, *p-adj* < 0.05) are shown by red dots above/outside dotted-line thresholds. **C** Top-most altered canonical pathways (*p* < 0.01) in FaTTy compared to parental cells obtained by Ingenuity Pathway Analysis. Only pathways with –2>z-score>2 are shown. **D**, **E** RT-qPCR data (mean + SEM) showing relative levels of selected transcripts associated with adipogenesis (**D**) and the cell cycle (**E**) in FaTTy cells (Early=PD72, Late=PD115-165) and parental MSCs (PD15). **F** Relative telomere length, indicated by T/S ratio (mean + SEM, please see Methods section) in parental MSCs and FaTTy across different PDs. **G** Representative brightfield images, together with nucleic acid content values (mean ± SEM, shown as Relative Fluorescence Units) of parental (PD20) and FaTTy (PD100) cells, as well as the tumour cell line, LNCaP, after 10 days of anchorage-independent growth in soft agar assay, showing distinct colony formation with an increase in nucleic acid content in LNCaP cells only. Scale bar = 100 µm. Significant mean differences (*p* ≤ 0.05) in D-G are denoted by different letters (abc).

We next sought to investigate the effects of immortalisation on genomic stability by comparing FaTTy with parental MSCs. G-banded analysis revealed minor aneuploidy, characterised by loss of Y chromosome, for FaTTy cells, while parental MSCs displayed a normal porcine karyotype (38, XY) (Fig. 2A). There was no indication of other large chromosomal translocation/re-arrangements associated with immortalisation.

Bulk RNA sequencing was performed using 3 biological replicates of non-differentiated FaTTy and progenitor cells. Results revealed a total of 572 upregulated and 708 downregulated transcripts in FaTTy compared to progenitor MSCs ( ≥ 2-fold change, *padj* < 0.05, Fig. 2B, Supplementary Data 1). QPCR validation of selected gene targets revealed a high degree of correlation with RNA sequencing results (Supplementary Fig. 4).

Gene enrichment analyses revealed, unsurprisingly, a predominance of lipid synthesis among significantly upregulated pathways in FaTTy cells (*P* < 0.01, Fig. 2C). Consistent with this, the expression of pro-adipogenic genes, such as *DPP4* (a marker of pre-adipocytes)^20,21^ and *FGF1*^22,23^, were expressed at substantially higher levels in FaTTy than in progenitor cells (Fig. 2D), whereas others such as *GATA3* and, particularly, *MEOX2*, two transcriptional inhibitors of adipogenesis^24–26^, were markedly downregulated, again keeping with FaTTy’s sustained adipogenic capacity in culture.

Interestingly, cell cycle checkpoints featured as the top downregulated pathway in FaTTy compared to progenitor cells (Fig. 2C), which is likely related to FaTTy’s ability to avoid replicative senescence during long-term culture^27^. To rule out the involvement of regulatory cell cycle genes that are commonly silenced or overexpressed in immortalised or transformed cells, we performed RT-qPCR on a subset of such genes, namely *RB1*^28^, *TP53*^29,30^, *MYC*^31^ and *TERT*^32^ (Fig. 2E). For all genes except *TERT*, differences between FaTTy and progenitor MSCs were either not detected or were comparatively small and inconsistent, suggesting a lack of causal involvement of those cell cycle regulators in FaTTy immortalisation. Expression of *TERT*, however, was consistently lower in FaTTy cells. Yet, quantification of telomere length across PDs (Fig. 2F) revealed a slight decrease in FaTTy relative to parental cells, but no significant changes during extended culture of FaTTy (PDs 82 to 160), suggesting telomerase-independent mechanisms for telomere length maintenance in FaTTy cells.

Next, we used a soft-agar colony formation assay to assess the tumorigenic potential of FaTTy (Fig. 2G). During the 10-day assay, FaTTy or parental MSCs were not able to form anchorage-independent colonies, whereas LNCaP cells (a human cancer cell line) did form colonies readily, in association with a distinct increase in nucleic acid content, indicative of their tumorigenic potential. Thus, although the mechanisms of FaTTy immortalisation have yet to be clarified, FaTTy does not display properties typical of malignantly transformed cells, thus encouraging its use in cultured food production.

### FaTTy gives rise to mature adipocytes showing marked lipid accumulation during long-term differentiation

**Fig. 3:**
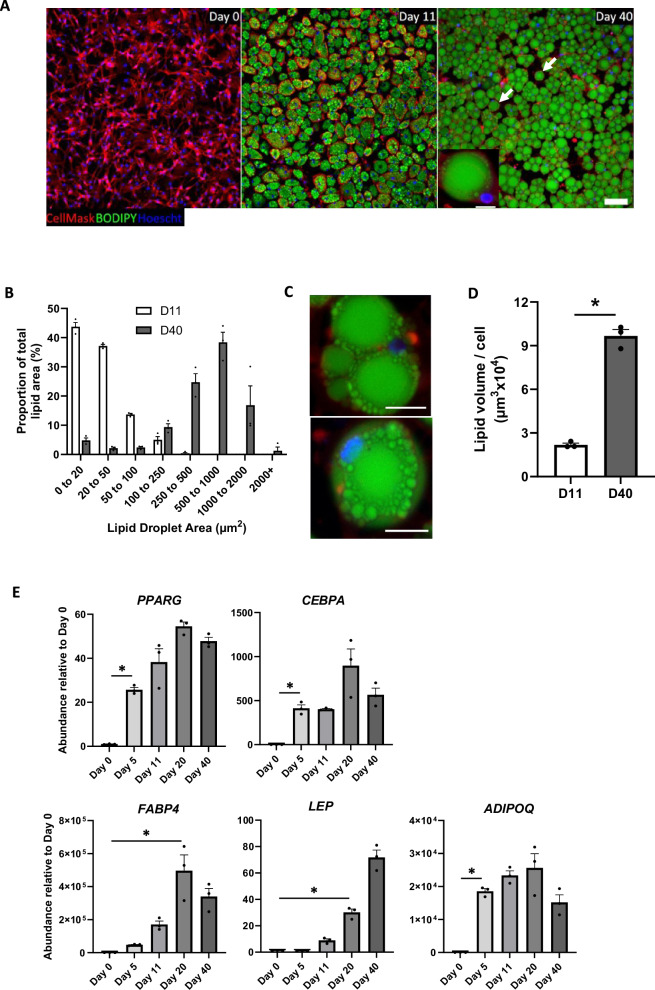
Image and gene expression analyses during long-term differentiation of FaTTy cells. **A** Representative confocal images of FaTTy cells (PD 115) taken on different days during a 40-day differentiation in 2D (scale bar = 100 µm). CellMask (red), Hoescht 33452 (blue) and BODIPY (green) stain cell membranes, nuclei and lipids, respectively. Inset in D40 image shows unilocular adipocyte (scale bar = 20 µm), as it is also indicated by white arrows in main image. **B** Frequency distribution of lipid droplet (LD) area as a proportion of total lipid area in FaTTy adipocytes at D11 and D40 of differentiation. **C** Representative confocal images of FaTTy-derived adipocytes showing ‘crowning’ of large LDs by small ones. Scale bar = 20 µm. **D** Mean lipid volume per cell in FaTTy adipocytes at D11 and D40 of differentiation (the star indicates a significant mean difference, *P* < 0.05). **E** Relative levels of selected transcripts over extended adipogenesis of FaTTy cells. For each transcript, the first significant increase in mean expression levels relative to Day 0 is shown by a star (*P* < 0.05).For all panels, where shown, bars represent mean + SEM.

To investigate the ability of FaTTy adipocytes to accumulate lipids over prolonged culture, cells were differentiated as per our standard protocol (see M&M section) followed by transfer to maintenance media without insulin from D11. Under these conditions, adipocytes changed their morphology from multilocular to paucilocular over the 40-day differentiation (Fig. 3A, Supplementary Fig. 5). Thus, adipocytes on D11 contained mostly small lipid droplets (LDs, area<100 µm^2^), whereas by D40 these had been replaced by much larger and fewer LDs (250 to 2000 µm^2^, Fig. 3B), signalling a shift towards the fusion and hypertrophy of LDs. In most adipocytes at D40, small LDs were observed to cluster around larger droplets, further suggesting fusion events (Fig. 3C). Moreover, when viewed as a Z-stack, the majority of small LDs resided at the base of the cell, with larger droplets expanding upwards and outwards (Supplementary Movie 1). Remarkably, unilocular adipocytes were detected on D40, but not D11, indicating acquisition of a mature white adipocyte phenotype during culture (Fig. 3A, Supplementary Fig. 5). In addition, average lipid volume per cell increased by 6-fold between D11 and D40 (Fig. 3D) to a maximum recorded of 96670 µm^3^, much higher than values reported so far in cultured cells^9^, and close to typical volumes of in-vivo adipocytes^33,34^.

To confirm the acquisition of a mature phenotype by FaTTy adipocytes, we quantified changes in the expression of early and late markers of adipogenesis at different time-points during the 40-day differentiation (Fig. 3E). We found that master transcriptional regulators, *PPARG* and *CEBPA*, were induced early on (by D5), as expected, and their expression peaked on D20. In contrast, mean expression levels of the late markers, *FABP4* and *LEP*, increased only by D20 and, in the case of *LEP*, continued to do so up until D40, thus correlating with increasing adiposity, as previously reported^35,36^. In addition, the levels of *ADIPOQ*, another late marker^37^ of adipocyte differentiation, increased as early as D5 and were maintained through D40. Thus, gene expression profiles confirmed that FaTTy produces adipocytes of mature phenotype.

### FaTTy adipocytes display similar fatty acid (FA) profiles to porcine adipose tissue but with an increased monounsaturated to saturated ratio

Ideally, lab-grown fat should have similar FA composition to animal-derived fat. Thus, we sought to establish FA profiles of FaTTy adipocytes relative to porcine adipose tissue and how they changed, if at all, during adipocyte maturation in vitro.

**Fig. 4:**
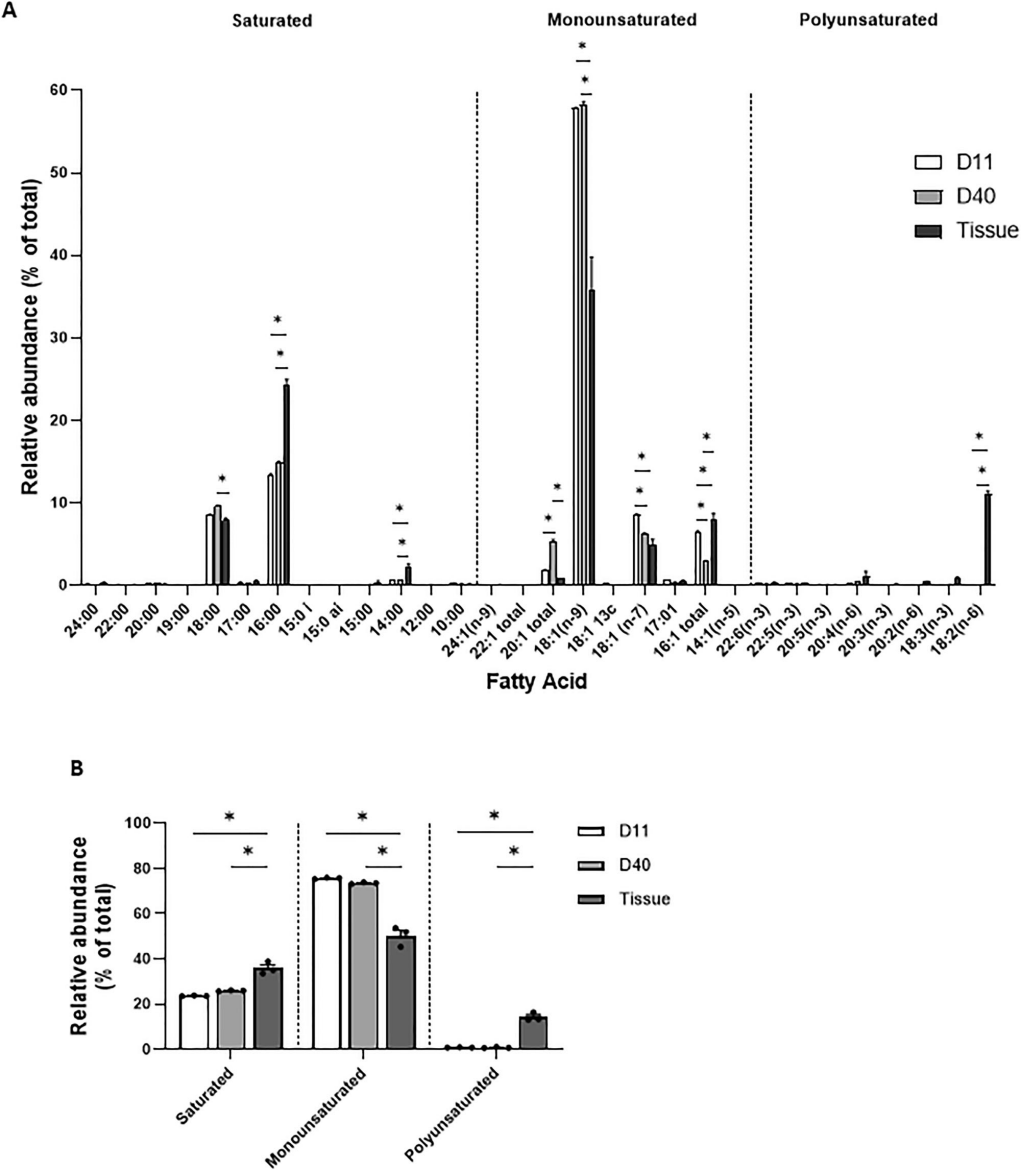
Lipid profiling of FaTTy adipocytes. Relative abundance of fatty acids, individually (**A**) and grouped by type (saturated, monounsaturated and polyunsaturated, (**B**) in FaTTy cells differentiated for 11 or 40 days, and in pig adipose tissue. Complete data can be found in Supplementary Data 2. Data is shown as mean + SEM, and significant differences between groups are shown by * (*p* ≤ 0.05).

FaTTy adipocytes at D11 and D40 were harvested and submitted for FA quantification by gas chromatography alongside subcutaneous fat samples collected from MSC donor piglets. Overall FA profiles were similar between in vitro-derived adipocytes and tissue samples (Fig. 4A, Supplementary Data 2), with a predominance, in both types of samples, of monounsaturated FAs (MUFAs) comprised mostly of oleic acid (18:1 n-9, 35.7–58.3% of total FAs across sample types), and saturated FAs (SFAs) comprised mostly of palmitic (16:0, 13.4–24.4% of total FAs) and stearic (18:0, 7.9–9.6% of total FAs). Proportionally, cultured cells contained less total SFAs and more total MUFAs than tissue, with total MUFA-to-total SFA ratios being 3.2, 2.8 and 1.4 for D11, D40 and tissue samples, respectively (Fig. 4B). This was largely accounted for by, respectively, reduced levels of palmitic and myristic (14:0), and higher levels of oleic in FaTTy adipocytes relative to tissue samples (Fig. 4A). Tissue samples also contained small levels of PUFAs (11% total FAs), almost exclusively in the form of linoleic acid (18:2, n-6). In contrast, cultured cells contained only negligible levels of linoleic acid (0.1%), as well as total PUFAs ( < 1%). Finally, the proportions of different types of FAs in cultured cells were, on average, similar between D11 and D40 (Fig. 4B), revealing that adipocyte hypertrophy was not associated with changes in overall FA profiles.

## Discussion

To realise the potential of cultured meat, it will be essential to have reliable cellular sources of its fundamental components. Fat is a key element, contributing significantly to the sensory and nutritional qualities of cultivated meat. Achieving industrial-scale production of cultivated fat will require well defined progenitor cell sources that can grow robustly in culture (ideally indefinitely) while maintaining the ability to differentiate into functional adipocytes reliably and with high efficiency. Moreover, lines devoid of genetic modifications are desirable and indeed strictly required for certain markets such as Europe and New Zealand^38^. All cell sources available from livestock species at present, including primary or immortalised MSCs, PSCs and DFAT cells, are far from fully meeting the above characteristics (reviewed in refs. ^3,39^). In the case of MSCs, a predominant cell source for cultured meat, gradual loss of differentiation capacity during passaging, alongside proliferative capacity, is a key limiting factor (see refs. ^7,8^ and Fig. 1). Here, we present a spontaneously immortalised pre-adipocyte line from pig, FaTTy, able to maintain relatively stable growth and, more impressively, close to 100% adipogenic efficiency together with the ability to generate mature adipocytes up to at least PD 200, demonstrating a much higher capacity than for previously reported highly adipogenic cell lines from livestock generated using genetic modification^15,40^. In addition, FaTTy could efficiently differentiate in absence of serum, and yielded FA profiles similar to native fat. Moreover, it showed no signs of genomic instability or tumorigenic potential, thus meeting many key requirements for a cell line appropriate for production of cultivated meat, and providing signficant potential as a source of in vitro fat for the industry.

To illustrate FaTTy’s capabilities we calculated, using the data above (Figs. 1B and 3E), that a single cell at PD70 expanded 70 times to PD140 would generate, following an 11-day differentiation, as much as 10^6^ tonnes of fat (considering a volumetric density for fat of 0.92 g/cm^3^), and 6 times that amount if cells were differentiated for 40 days. This, together with FaTTy’s capacity for sustained expansion and differentiation in culture further demonstrates its considerable potential for meeting the high demands of the cultivated meat industry. Yet, all data in our study were obtained after expansion of FaTTy cells in 2D in the presence of serum; a key requirement for industrial scalability will be adaptation of the cell line to suspension culture, to allow for growth in bioreactors, in addition to growth in serum-free conditions^16^.

Although genomic analyses did not provide substantial clues as per the mechanisms behind FaTTy’s emergence in culture, or its extreme adipogenic phenotype, the noted gain of adipogenic potential during culture together with its aneuploidy pattern (loss of Y chromosome) suggests FaTTy may have originated clonally from a distinctly adipogenic subpopulation within progenitor cells that gained a growth advantage in culture. Indeed, FaTTy progenitors contained a distinctly higher proportion of committed pre-adipocytes compared to other MSC lines tested, as demonstrated by a close to 50% differentiation efficiency of MSC5 cells (Fig. 1D); in this context, loss of Y chromosome could have contributed to conferring a distinct growth advantage to those cells^41,42^. Of interest, despite a dramatic increase in the adipogenic capacity of FaTTy, maintained under a variety of culture conditions, expression of the two adipogenic master regulators, *PPARG* and *CEBPA*, did not change or was actually lower (for *CEBPA*) in FaTTy pre-adipocytes compared to progenitor cells (Supplementary Data 1), underscoring the complexity of the mechanisms underlying FaTTy’s unique phenotype and warranting additional investigation.

Maturation of white adipocytes in vivo is characterised by significant hypertrophy and fusion of cytoplasmic lipid droplets to form paucilocular or unilocular adipocytes^43^. In vitro, adipogenesis leading to unilocular, mature adipocytes has been reported in several 3D systems^44,45^, whereas differentiation in 2D typically results in multilocular adipocytes representing immature, foetal-stage phenotypes^46^. Conceivably, routine protocols for adipocyte differentiation may be too short for appropriate lipid droplet fusion to occur in 2D contexts. In a recent study, porcine adipocytes generated from DFAT cells were maintained for as long as 30 days, however, unilocular cells were not produced, even with FA supplementation^47^. In contrast, after long-term maturation in culture, FaTTy cells displayed substantial hypertrophy and produced mature unilocular adipocytes, the first ever such observation in a livestock species. This observation further attests to the unique capabilities of FaTTy as it relates to production of fat in vitro, as well as its considerable potential as research tool for studying adipocyte biology in livestock.

We found that FA profiles were largely similar between FaTTy adipocytes and native pig fat. However, a distinct feature of FaTTy adipocytes was that they contained negligible levels of PUFAs (Fig. 4), a finding that could be attributed to a deficiency of PUFA precursors in media used for differentiation^47^, and that is a limitation in the present study. Of note, FaTTy adipocytes also displayed about 2-fold higher MUFA-to-SFA ratios than native pig fat, a phenomenon not reported before in in vitro-derived adipocytes. Diets with high MUFA to SFA ratios have been implicated in improving cholesterol levels and heart health, among other benefits^48–50^. Thus, our results indicate that cultivated pig fat may potentially provide a healthier alternative to traditional fat. Yet, in practice, differences in the FA profiles of in vitro-derived adipocytes may affect other qualities such as flavour^51^, potentially compromising their palatability as food components. In this regard, the opportunity to tailor FA profiles in cultured adipocytes to specific consumer needs, either through culture media supplementation^12,47^ or gene engineering approaches^52^, holds significant promise in the field of animal-free designer foods.

In conclusion, FaTTy is a unique livestock cell line with distinct adipogenic phenotype characterised by the ability to reliably differentiate with high-efficiency under a variety of culture conditions, and to generate mature adipocytes displaying fatty acid profiles comparable to native fat. These features, together with its non-GMO nature, make FaTTy a highly promising foundational tool that, provided it is adapted to expansion in suspension-based, serum-free culture, could contribute to making culture meat sustainable as an alternative to animal farming and provide greater food security to future generations.

## Methodology

### Cell culture

MSC populations were derived in a previous study from subcutaneous adipose tissue samples of 5 Large White x Landrace male piglets (21 days old or younger), as described^53^. MSCs were grown in DMEM High Glucose (Gibco, 41965039) supplemented with 10% FBS (Gibco, A5256801), 1% Penicillin Streptomycin (Gibco, 15140-122) and 5 ng/ml human basic fibroblast factor (bFGF) (PeproTech, AF-100-18B-1MG) on plasticware precoated with 0.1% bovine gelatine (Merck, 9000-70-8) at 39^o^C and 5% CO_2_. Media was changed every 48 h and cells were passaged at 70-90% confluence using Trypsin-EDTA (Gibco, 25200-056). Seeding density was increased from 2000/cm^2^ to 4000 cells/cm^2^ after 100 population doublings. FaTTy cells were assessed for mycoplasma during expansion (Supplementary Fig. 6), using Venor® GeM Classic kit (Merck, MP0025) according to manufacturer’s instructions.

LnCaP cells (kindly provided by Dr. Jenny Fraser, R(D)SVS, The University of Edinburgh) were cultured under same conditions as pig MSCs, but on uncoated wells.

Population Doubling Times (PDTs) were calculated as per the formula below, with rounding to the nearest quarter hour;

$$PDT=\frac{Time\,(h)\times {\rm{l}}{\rm{n}}\,(2)}{{\rm{l}}{\rm{n}}\,(\frac{Final\,no.cells}{Initial\,no.cells})}$$

Population doublings (PDs) were calculated by dividing time in culture by average PDT calculated over consecutive ranges of time in culture. Passage number equivalence for each PD is provided in Supplementary Data 4.

### Adipogenic differentiation in 2D and image analyses

Cells (20,000/cm^2^) were seeded onto wells pre-coated with 0.1% gelatine and grown to confluence, after which (Day 0) growth medium was exchanged to standard differentiation media (DMEM High Glucose with 10% FBS and 1% Penicillin-Streptomycin) to which an inducer cocktail was added consisting of 0.5 mM IBMX (Sigma-Aldrich, 41095), 1 µM dexamethasone (Sigma-Aldrich, D4902), 1.8 µM insulin (BioXtra, I9278-5ML), and 100 µM Indomethacin (STEMCELL technologies, 73942). Media was refreshed after 72 h, and on Day 5 (or Day 3 for rapid differentiation experiments) it was changed to maintenance media consisting of standard differentiation media supplemented with 1.8 µM insulin, refreshed every 48 h. For long term differentiation (40 days), insulin was removed from maintenance media from Day 11, with media exchanged every 2-3 days.

In some experiments, standard differentiation media was used without FBS (serum-free), or with FBS replaced by 10% KnockOut Serum Replacement (Gibco, 10929010), or chemically defined media (modified from Kolkman, et al.^54^) consisting of 1% ITS-X (Gibco^TM^, 51500056), 1% Glutamax (Thermo Fisher Scientific, 35050061), 1 µg/ml α-linoleic acid (Thermo Fisher Scientific, 30282500), 36 ng/ml Hydrocortisone (Sigma-Aldrich, H0888), 20 ng/ml IL-6 (Peprotech, 200-06), 10 ng/ml bFGF and 10 ng/ml PDGF-BB (Peprotech, 100-14B).

Differentiated cells were fixed in 4% PFA (VWR chemicals, VWR-P38-Sh) and stained with BODIPY™ 493/503 (4 mM, 1:2500, Invitrogen, D3922), Hoescht 3342 (1 mg/ml, 1:1500, Invitrogen, H21486), and CellMask Red (1:1250, Invitrogen C10046) for 15 min before imaging using Zeiss Cell Observer (Zeiss, Germany). Four representative images from quadruplicate samples/wells were captured using Axio Observer.Z1/7 Microscope (Objective: EC Plan-Neofluar 10x/0.30 Ph1 magnification). Adipocytes were defined as distinct cells containing BODIPY positive lipid droplets, and differentiation efficiency was defined as number of adipocytes/total nuclei. Both adipocytes and nuclei were analysed manually using ZenBlue imaging software (version 3.1). CellMask Red was used to define cell bodies where unclear.

For analysis of lipid droplets, stained cells were imaged using Zeiss LSM 880 Confocal Laser Scanning Microscope (Zeiss, Germany). Lipid droplets were defined as areas positive for BODIPY. Lipid droplet area was calculated using AdipoQ software^55^ via Fiji using images of a single focal plane. Lipid droplet volume was calculated by analysing Z-stacks of cell populations via IMARIS software. Machine learning based object classification was applied batchwise on Day 11 and Day 40 images, respectively, following training on respective datasets, to calculate lipid volume as sum of objects. Total lipid volume per cell was calculated as the sum of lipid droplet volume divided by number of nuclei present in a particular Z-stack.

### Differentiation in sodium alginate hydrogel

Cells were resuspended in 0.5% sodium alginate solution (Merck, W201502) at a concentration of 3 × 10^7^/ml, as described^9^. Cell mixtures were inserted via 20 ul pipette tip into CaCl2 solution by a singular, sweeping pipette motion to produce alginate ‘strings’, which were incubated for 15–20 min at 39 °C to cross-link and then washed twice with PBS. Fifteen µl of cell mixture (450,000 cells) were used per well in 24 well plates and fed with 1.5 ml media.

Cells in hydrogel were kept on a Grant Bio CPS-20 orbital shaker at 70 rpm and immediately differentiated for five days with adipocyte differentiation media, then changed to adipocyte maintenance media, with media refreshed every 48 h. Hydrogels were stained with BODIPY as detailed above without fixation, and imaged with Zeiss LiveCell Imager.

### Lipidomic analysis

Cells differentiated in triplicate 12-well plates for either 11 or 40 days were washed twice with DPBS (Gibco, 14190144), then collected with a 20 mm cell scraper (Starlab, CC7600-0300) in 200 ul DPBS. Lipidomic analysis was performed by MyInfied Lipid Analysis (James Hutton Ltd., Dundee, UK). In brief, after freeze-drying the samples to remove any moisture, fatty acids were trans-esterified in situ with 1.5 N HCl in methanol in the presence of toluene. The resultant fatty acid methyl esters (FAMEs) were extracted with toluene, separated, identified, and quantified by gas chromatography (GC) using a Cp-Select for FAME GC column (200 m × 0.25 mm internal diameter. x 0.25 mm film thickness, Agilent Technologies Part No. CP7421).

### Soft agar assay

Soft agar assay was performed using CytoSelect™ 96-Well Cell Transformation Assay, Soft Agar Colony Formation kit (Cambridge Bioscience, CBA-130) as per manufacturer’s protocol. Cells (10,000/well) were seeded in quadruplicate, grown for 11 days and then imaged using an Axiovert 5 microscope. Nucleic acid was isolated and incubated with CuQuant GR Dye, then assessed using BioTek synergy HTX reader (excitation 485/20, emission 528/20, automatic scalings). LNCaP cell line was used as a positive control.

### Karyotype analysis

Karyotyping was performed by Creative Bioarray (Sherley, NY, USA). Colcemid Solution (Thermofisher #15210-040) was added to the cell cultures at a final concentration of 0.5 ug/ml, and incubated at 37 °C, 5% CO_2_ for 3 h. Cells were transferred to 15 ml centrifuge tubes, and spun at 300 g for 7 min, then resuspended in 0.075 M KCL hypotonic solution. After 7 min at room temperature cells were spun at 300 g for 7 min, and resuspended in 3:1 methanol: acetic acid fixative. After 10 min the fixed cell suspensions were centrifuged as above. Cell pellets resuspended in 0.5 ml of fixative were used to create metaphase chromosome spreads by dropping a single drop of suspension onto each distilled water-soaked microscope slide. Slides were baked at 90 °C for 50 min, treated with 0.1% trypsin-EDTA, and stained with Wright’s Giemsa stain in Gurr’s Buffer at pH 6.8. Metaphase chromosome spreads were analysed by brightfield microscopy at 1000X utilizing Leica Biosystems CytoVision karyotyping software (version 7.7).

### RNA-sequencing

Cell samples were trypsinised, washed twice with PBS, resuspended in TRIzol reagent (Invitrogen, 15596026), and stored at –80 °C. For RNA extraction, cells homogenized in TRIzol and 1-bromo-3-chloropropane (Sigma-Aldrich, B62404**)** were incubated at room temperature for 3 min then centrifuged for 15 min (12,000 × *g* for 4 °C). Aqueous layers containing RNA were removed and purified using RNeasy Mini kit (Qiagen, 74104) as per manufacturer’s instructions.

RNA sequencing was performed by Genewiz UK, Azenta Life Sciences (Takeley, Essex, UK). Briefly, the RNA sequencing library was prepared using the NEBNext Ultra II Directional RNA Library Prep Kit following manufacturer’s instructions (New England Biolabs), then validated using DNA Kit on an Agilent 5600 Fragment Analyzer (Agilent Technologies) and quantified on a Qubit 4.0 Fluorometer (Invitrogen). The library was then sequenced using the Illumina NovaSeq 6000 platform according to manufacturer’s instructions using a 2 × 150 bp Paired End configuration (v1.5). NovaSeq Control Software (v1.7) was used to perform image analysis and base calling. Raw sequencing files were converted into fastq files and de-multiplexed using Illumina bcl2fastq (v2.20) software, allowing one mismatch for index sequence identification.

Quality control of the raw data was performed using FastQC, after which adaptor sequences and poor-quality nucleotides were removed using Trimmomatic (v0.36). Trimmed reads were mapped to the reference genome Sus scrofa 10.2 (Ensembl) using the STAR aligner (v2.5.2b). Counts were calculated using the Subread package (v1.5.2) Counts feature, only including unique reads within exons.

Differential expression analysis was performed using DESeq2-Bioconductor software (v1.16.1). Data was analysed, processed, and visualised using standard R coding (v4.3.3) with packages dplyr (v1.1.4), ggplot2 (v3.5.0), ggrepel (v0.9.5), ggthemes (v5.1.0), pheatmap (v1.0.12), RColorBrewer (v1.1-3), rlog (v0.1.0), and tidyverse (v2.0.0). Data were further analysed with QIAGEN IPA software (QIAGEN Inc., https://digitalinsights.qiagen.com/IPA), using DEGs with absolute fold change ≥ 2 and *p-adj* < 0.05.

### RT-qPCR

For cDNA synthesis, 300 ng RNA were incubated with 0.5 µl random primers (250 ng/µl, Promega, 1181), 1 µl dNTPs (10 mM, Invitrogen, 18427-013) and nuclease-free water in a thermocycler (Biometra) at 65 °C for 5 min, followed by 5 min at 4 °C. Then, 1 µl DTT (0.1 M, Invitrogen, 18080-93), 1 µl RNasin Plus RNase inhibitor (40 U/µL, Promega, N2611), 1 µl SuperScript III reverse transcriptase (Invitrogen, 18080-93), and 4 µl first strand buffer (5X, Invitrogen, 18080-93) were added and samples incubated at 25 °C for 5 mins, followed by 50 °C for 60 min, 70 °C for 15 mins and a cooling step at 4 °C for 5 min. No-template and no-RT-controls were included. cDNA was kept frozen at −20 °C.

cDNA samples (2 µl, 1/40 dilution, two technical duplicates) were combined with 5 µl SensiFAST SYBR green (Meridian Bioscience, BIO94020), forward and reverse primer (10 µM, 0.4 µl each, Supplementary Data 3), and 2.2 µl nuclease-free water, and subjected to 95 °C for 2 min, followed by 40 cycles of 95 °C for 5 s, 60 °C for 11 s and 72°C for 5 s, and a final incubation at 95 °C for 1 min, 60 °C for 30 sec and 95°C for 30 s in an AriaMx Real-Time PCR machine (Agilent). Target gene copy numbers were calculated using a standard curve made up of 4-fold dilutions of a pool of cDNA, and normalisation to copies of the housekeeping genes, *RPL4* and *TOP2B*, within each sample. Data were analysed using Agilent Aria 2.1 software.

### Quantification of relative telomere length

Cell pellets were prepared from half million growing cells and stored at −80°C. DNA extraction was performed using a Monarch® Genomic DNA Purification Kit (NEW ENGLAND Biolabs, T3010S) according to manufacturer’s instructions. For QPCR, DNA was processed as described above and subjected to 95 °C for 2 min, followed by 40 cycles of 95 °C for 5 s, 56 °C for 10 s and 72 °C for 20 s, and a final incubation at 95 °C for 1 min, 60 °C for 30 s and 95 °C for 30 s in an AriaMx Real-Time PCR machine (Agilent). The relative telomere (T) copy number thus obtained was normalized to the relative copy number of a single copy (S) gene (*GUSB*) generating a T/S ratio which was used to indicate telomere length^56^.

### Statistics

All experiments were performed using at least biological triplicates. Graphing and statistical analysis were performed in GraphPad 10 or R (v.4.3.3). Data normality was confirmed using a Shapiro-Wilk test. Data were analysed by one-way or two-way ANOVA with Tukey’s multiple comparison test, or unpaired t-test when only two means were being compared.

## Supplementary information

Supplementary Data1

Supplementary Data2

Supplementary Data3

Supplementary Data4

Supplementary Movie1

Supplementary information

## Data Availability

Raw fastq files and counts from RNA sequencing were deposited in NCBI’s GEO under Series GSE266992. All other data is available within the manuscript.
