# Publisher Correction: A unique spontaneously immortalised cell line from pig with enhanced adipogenic capacity

**DOI:** 10.1038/s41538-026-00780-0

**Published:** 2026-03-11

**Authors:** Thomas Thrower, Susanna E. Riley, Seungmee Lee, Cristina L. Esteves, Donadeu F. X.

**Affiliations:** Division of Translational Bioscience, The Roslin Institute and Royal (Dick) School of Veterinary Studies, University of Edinburgh, Edinburgh, UK

**Keywords:** Mesenchymal stem cells, Fats, Cell growth

Correction to: *npj Science of Food* 10.1038/s41538-025-00413-y, published online 20 April 2025

“In this article, the author’s name ‘Donadeu F.X.’ was incorrectly written as ‘Xavier Donadeu F’. The original article has been corrected.”

