# Supplementary material for: A unique spontaneously immortalised cell line from pig with enhanced adipogenic capacity
Source: NPJ Sci Food. 2025 Apr 20;9:52. doi: 10.1038/s41538-025-00413-y (PMC12010005; doi:10.1038/s41538-025-00413-y)
Supplement: Supplementary file 6 — Supplementary information [file 41538_2025_413_MOESM6_ESM.pdf]

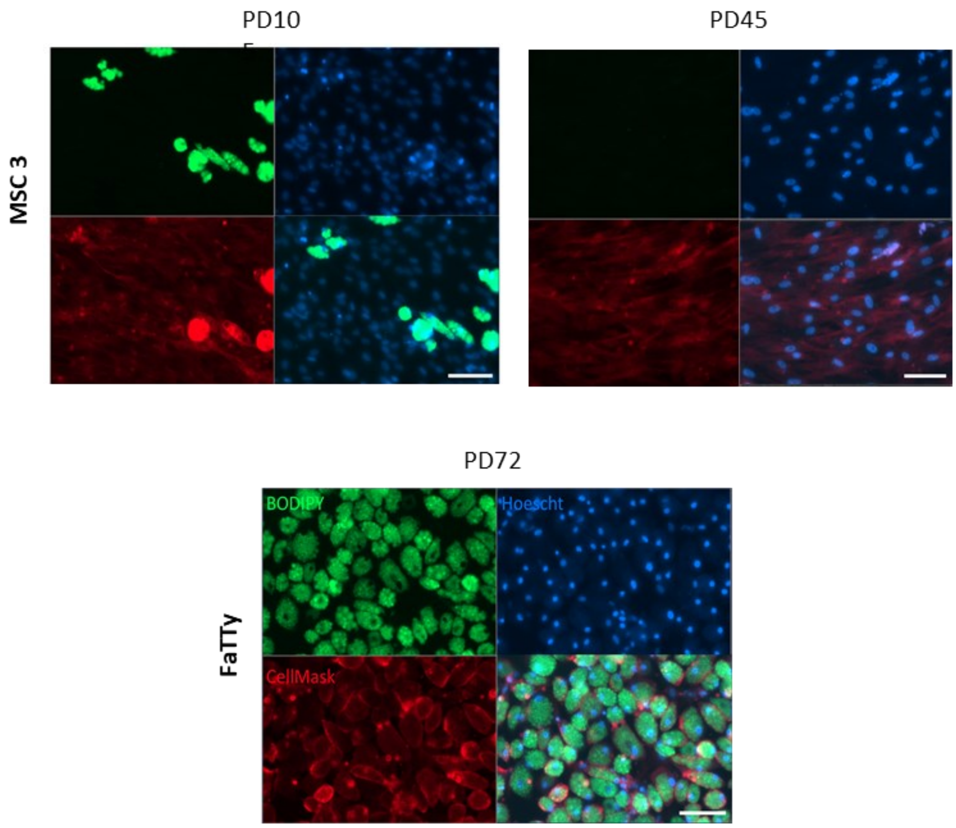

Supplementary Figure 1. Differentiation efficiency of all MSC lines tested, except FaTTy, decreased during serial passaging. Representative fluorescent adipocytes images obtained from MSC3 cells at early and late PDs, and from FaTTy, following incubation with adipogenic media. Green = lipid (BODIPY); Blue = nucleus (Hoescht 33452).

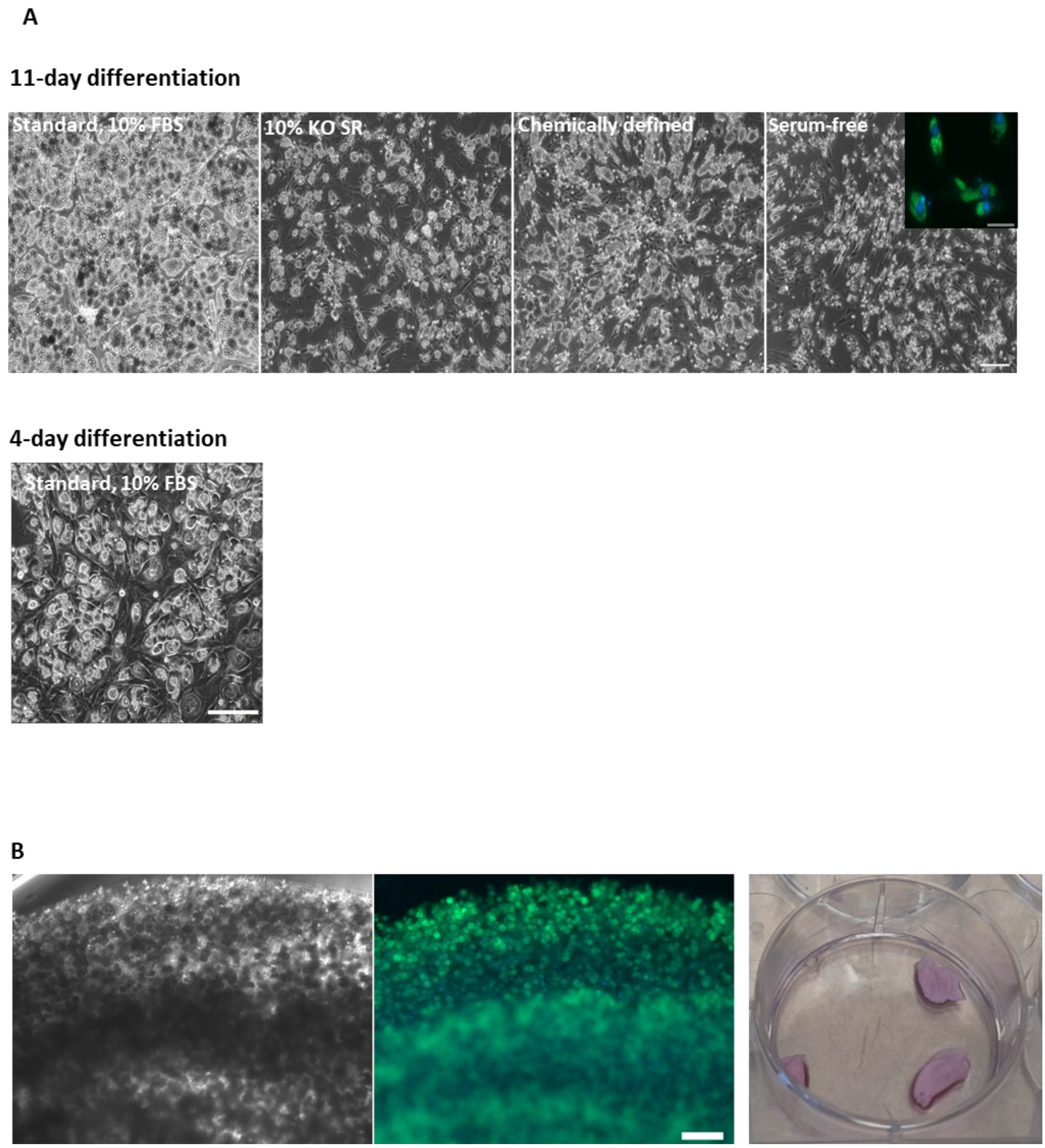

Supplementary Figure 2. Efficient differentiation of FaTTy cells under a variety of conditions. (A) (Top row) Representative brightfield images of adipocytes obtained after differentiation of FaTTy cells for 11 days using standard serum-containing media or media where serum had been removed or replaced with KO serum replacement or chemically defined media, as detailed in Methods (scale bar = 100 μm). Inset shows presence of lipid-filled adipocytes even in the absence of serum (lipid and nuclear stains shown by green (BODIPY) and blue (Hoescht 33452), scale bar = 20 μm), demonstrating capacity of FaTTy-

derived adipocytes for *de novo* lipogenesis. (Bottom row) Image of adipocytes obtained after differentiation of FaTTy cells for four days using standard serum-containing media. Scale bar = 50 μm. (B) Representative brightfield (left) and fluorescence (middle, stained with BODIPY) images denoting a high degree of adipocyte accumulation after differentiation of FaTTy cells in an alginate hydrogel. Scale bar = 100 μm. 3D 'steaks' of fat produced after differentiation in hydrogels are also shown (right).

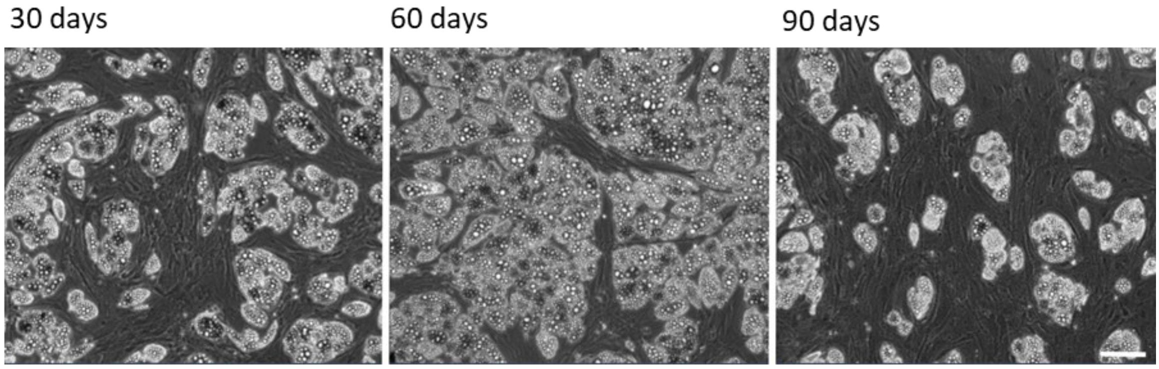

Supplementary Figure 3. Further attempts to re-derive FaTTy-like cells from MSC5 progenitors did not yield cells with sustained differentiation capacity. Representative brightfield images showing a reduction in the ability of MSC5 cells to form adipocytes upon continuous expansion for about 30, 60 or 90 days. In each case, Images were taken after an 11-day differentiation (scale bar = 100 μm).

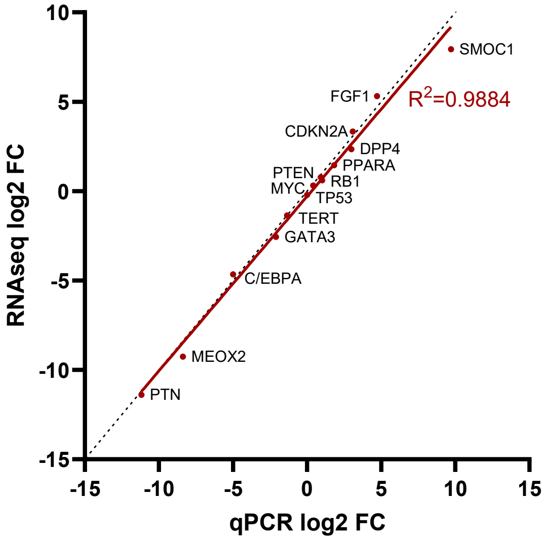

Supplementary Figure 4. Correlation between RNA-seq and RT-qPCR data. Comparison of the log2 fold changes in selected transcript abundance between FaTTy and its parental cell line as measured by bulk RNA-sequencing and RT-qPCR. R squared value was calculated by simple linear regression. Dotted line denotes y=x.

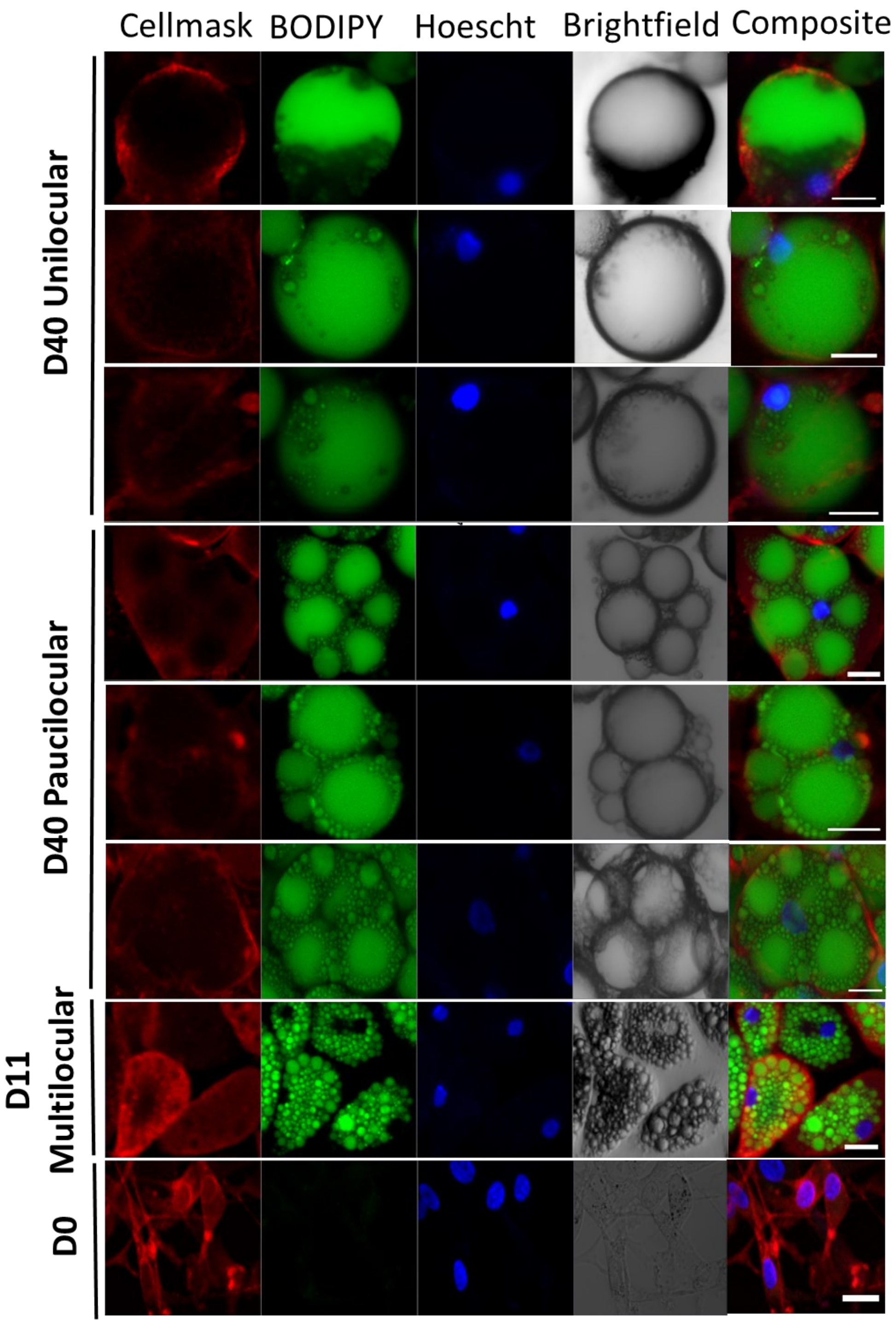

Supplementary Figure 5. Morphology of FaTTy-derived adipocytes. Representative images of individual adipocytes obtained after differentiation of FaTTy cells for 11 or 40 days, illustrating multilocular, paucilocular and unilocular phenotypes. Undifferentiated FaTTy cells (D0) are also shown. CellMask (red), Hoescht 33452 (blue) and BODIPY (green) stain cell membranes, nuclei and lipids, respectively. Scale bar = 20µm

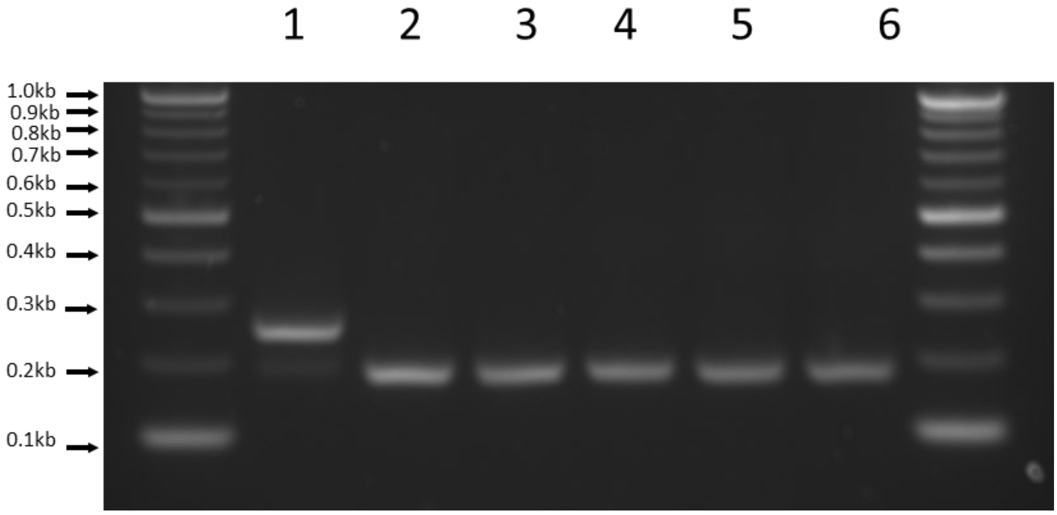

Supplementary Figure 6. Mycoplasma screen. Representative mycoplasma screen, visualised by agarose gel. Lanes from left to right, 1: positive control reaction (expected band size, 265-278 bp), 2: negative control reaction (expected band size, 191 bp), 3, 4: FaTTy PD150, 5: MSC1, 6: FaTTy PD 110. DNA ladder is shown at either side.

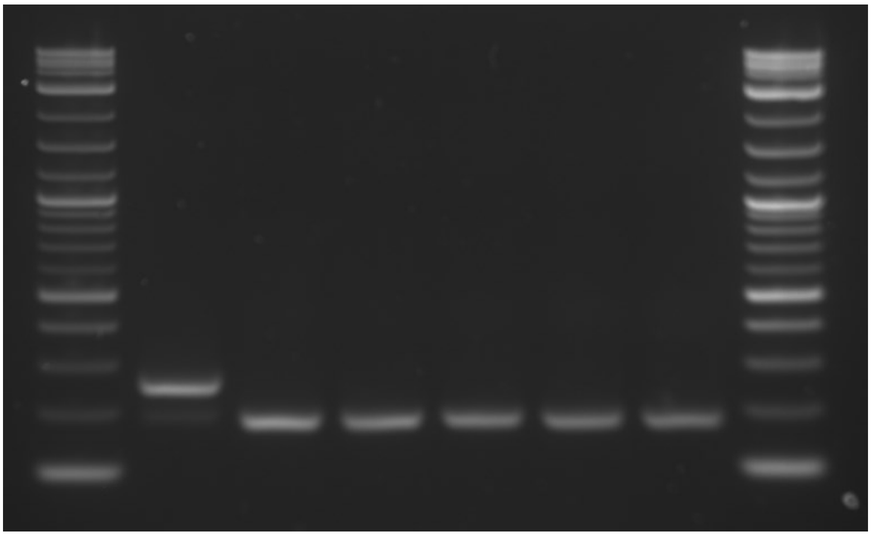

Supplementary Figure 6 uncropped and unprocessed.

Supplementary Movie 1. 3D Imaging of FaTTy-derived adipocytes. Lipid droplets of FaTTy-derived adipocytes grown for 40 days visualized via a z-stack of 37 slices of 0.99 µm. Green = lipid; Blue = nucleus; Red = cell membrane.
